# Periodontal Disease and Heart Failure: Current Evidence and Perspectives

**DOI:** 10.1007/s11886-026-02438-5

**Published:** 2026-10-17

**Authors:** Alícia Eliege da Silva, Valéria Duarte de Almeida, Gilson Aquino Cavalcante, Roque Ribeiro da Silva Júnior, Daria Raquel Queiroz de Almeida, José Veríssimo Fernandes, Ellany Gurgel Cosme do Nascimento, Cléber de Mesquita Andrade, Thales Allyrio Araújo de Medeiros Fernandes

**Affiliations:** 1 Universidade do Estado do Rio Grande do Norte, Mossoró, RN Brazil; 2 Universidade Federal do Rio Grande do Norte, Natal, RN Brazil

**Keywords:** Periodontal disease, Periodontitis, Heart failure, Systemic inflammation, Cardiac biomarkers, Oral-systemic health

## Abstract

**Purpose of Review:**

Periodontal disease has been increasingly recognized as a chronic inflammatory condition with potential systemic implications, particularly in the cardiovascular system. Heart failure (HF), a pro-inflammatory syndrome with multisystem involvement, remains a major public health burden. This state-of-the-art narrative review aims to analyze the current scientific evidence on the association between periodontal disease and heart failure, emphasizing epidemiological data, cardiac biomarkers, inflammatory mediators, and future perspectives.

**Recent Findings:**

Current evidence indicates a high prevalence of periodontal disease among patients with HF and suggests associations between periodontal disease and elevated cardiac biomarkers, including NT-proBNP and troponin T, as well as systemic inflammatory mediators such as C-reactive protein, IL-6, and TNF-α. Interventional studies have reported reductions in some of these biomarkers following periodontal treatment, suggesting a potential biological link between periodontal inflammation and HF. However, findings remain heterogeneous, and some studies have failed to demonstrate significant associations.

**Summary:**

Current evidence supports an association between periodontal disease and heart failure, with systemic inflammation representing a potential biological link. Nevertheless, the heterogeneity of study designs, small sample sizes, and predominance of observational and cross-sectional evidence limit causal inference. Longitudinal studies and randomized clinical trials are needed to clarify causal mechanisms and determine whether periodontal prevention and treatment can contribute to integrated strategies for patients with HF.

## Introduction

Heart failure (HF) is an increasing challenge for public health, affecting approximately 37.7 million people worldwide [1, 2]. Frequently described as the final stage of various cardiovascular diseases, its rising incidence is a matter of concern due to the significant impact on patients’ quality of life, who often present with symptoms such as dyspnea, fatigue, exercise intolerance, and fluid retention [3]. Despite recent therapeutic advances, mortality rates associated with HF remain high, with approximately half of the patients dying within five years after the initial diagnosis [4, 5].

Periodontitis is a chronic inflammatory disease and one of the leading causes of tooth loss [6]. This condition negatively affects the quality of life of affected individuals, causing functional impairments in mastication, swallowing, and speech, in addition to compromising oral aesthetics [4, 7]. Chronic inflammation arises from the complex interaction between the microbial biofilm and the host immune response, with periodontal pathogens capable of modulating immunity both locally and systemically through the production of endotoxins. These endotoxins stimulate the release of pro-inflammatory cytokines, which may amplify and perpetuate inflammation in distant tissues, including the cardiovascular system [8–10].

HF is characterized by a pro-inflammatory state that manifests not only in the heart but also in the blood vessels and other organs, with prevalent multisystem involvement [11, 12]. Chronic systemic inflammation associated with venous congestion is a frequent finding in patients with HF [13, 14]. Conversely, individuals with periodontitis present with oral microbial dysbiosis, which is closely linked to increased levels of systemic inflammation [15, 16]. A comprehensive meta-analysis of randomized clinical trials demonstrated significant reductions in C-reactive protein (CRP), an important inflammatory marker, following periodontal treatment [5, 10, 17].

Given the evidence suggesting a relationship between chronic systemic inflammation triggered by periodontitis and its implications for cardiovascular health, it is essential to deepen the understanding of this link, particularly in the context of heart failure, a condition with high morbidity and mortality. Although several studies have reported an association between periodontal health and cardiac function, the underlying pathophysiological mechanisms remain under debate, and the available findings still exhibit heterogeneity. Therefore, the present study aims to critically analyze the current scientific literature on the association between periodontal disease and heart failure, discussing the main evidence, proposed biological mechanisms, and existing gaps that may guide future research.

### Prevalence of Periodontitis in Patients with Heart Failure

Periodontitis is a chronic inflammatory disease that affects gingival tissues, periodontal ligaments, and alveolar bone, and represents one of the leading causes of tooth loss. Beyond its local consequences, periodontitis has been associated with several systemic conditions, particularly cardiovascular diseases [18].

Evidence suggests that patients with coronary artery disease (CAD), one of the main causes of chronic heart failure (CHF), show a higher prevalence of periodontitis, whereas individuals with periodontitis are at increased risk of CAD, myocardial infarction, and stroke, all of which are recognized as important contributors to CHF. This association between periodontitis and adverse cardiovascular outcomes has been reinforced by international consensus reports, such as those by Tonetti and Van Dyke and Dietrich et al., which highlight periodontitis as an independent risk factor for atherosclerotic disease [19, 20]. Therefore, it is plausible to hypothesize a relationship between periodontitis and CHF [21].

Population-based studies further reinforce this association. Using data from the Third National Health and Nutrition Examination Survey (NHANES III), Yan et al. reported that individuals with moderate to severe periodontitis had a significantly higher prevalence of CHF compared with those without periodontitis or with only mild forms of the disease [22]. Similar findings were reported by Aoyama et al., who observed a higher rate of CHF among patients with elevated antibody levels against periodontal pathogens such as *P. gingivalis*, *A. actinomycetemcomitans*, and *P. intermedia*, supporting the hypothesis that the immune response to periodontal infection may contribute to cardiac pathophysiology [23].

Other investigations corroborate this pattern. Schmalz et al. demonstrated a high prevalence and severity of periodontitis in cohorts of patients with chronic heart failure (CHF), additionally linking periodontal inflammation parameters to a higher incidence of systemic infections [24]. Similarly, Ziebolz et al. reported that most patients with cardiomyopathy, whether of ischemic or non-ischemic dilated origin, presented with severe periodontitis [25]. Comparable findings had previously been described by Fröhlich et al., who observed that none of the CHF patients examined had completely healthy gingival tissue, regardless of the etiology of heart failure [21].

Taken together, these findings indicate that the prevalence of periodontitis among individuals with CHF is consistently high, regardless of factors such as age, etiology of cardiomyopathy, or the presence of comorbidities. This suggests that periodontal disease may be implicated not only as a risk marker but also as a potential factor in the progression of heart failure.

### Association Between Periodontitis and Cardiac Biomarkers

Neurohormonal activation is a central component of the pathophysiology of HF, reflected in elevated levels of natriuretic peptides, particularly the N-terminal pro–B-type natriuretic peptide (NT-proBNP). This biomarker is widely used in the diagnosis and management of HF because of its ability to reflect both hemodynamic overload and clinical severity of the disease [26]. In recent years, several studies have suggested that the presence of periodontal disease (PD) may be associated with increased levels of cardiac biomarkers, especially NT-proBNP.

In a multicenter cohort [5], Molinsky et al. observed that individuals with periodontitis or edentulism had higher mean NT-proBNP levels compared with those with a healthy periodontium, even after adjustment for confounding variables. Similar results were reported by Schulze et al., who found significantly higher concentrations of NT-proBNP and troponin T in HF patients with periodontitis compared with those without the periodontal disease [10].

In line with these findings, Walther et al. demonstrated that patients with severe periodontitis not only exhibited higher NT-proBNP levels but also greater elevations of circulating inflammatory biomarkers such as CRP and interleukin-6. Moreover, they observed that the association between severe periodontitis and heart failure was particularly evident in individuals with reduced or mildly reduced ejection fraction, suggesting that periodontal inflammation may be more strongly linked to forms of HF characterized by greater ventricular dysfunction [27].

Additional evidence strengthens this relationship: systematic reviews have shown that periodontitis is associated with persistently elevated levels of CRP, a key marker of systemic inflammation [28], and clinical studies have documented endothelial dysfunction in patients with periodontal disease, a condition that may represent a potential link between oral inflammation and cardiac remodeling [29, 30].

However, the results in the literature are not entirely consistent. Dekker et al., when comparing serum and salivary levels of B-type natriuretic peptide (BNP) in HF patients, found no significant correlation between these parameters, suggesting that the biomarker may vary according to the biological fluid analyzed [31]. Similarly, Aoyama et al. (2019) did not identify statistically significant differences in BNP levels between patients with high and low antibody titers against *Porphyromonas gingivalis* [23].

On the other hand, longitudinal studies provide indications of a more robust causal link. Czerniuk et al. followed patients with CHF and periodontitis who underwent periodontal treatment and observed a statistically significant reduction in plasma NT-proBNP concentrations after six months, suggesting that improvement in periodontal health may positively influence cardiac biomarkers and, potentially, the clinical course of HF [26].

Taken together, these findings suggest that periodontal disease may contribute to the elevation of cardiac biomarkers in patients with HF, although controversies remain due to methodological differences, diagnostic criteria, and the specific biomarkers analyzed. Nevertheless, the growing body of evidence highlights the relevance of periodontal health as a potential modulator not only of the inflammatory profile but also of the neurohormonal profile in these patients.

### Periodontal Disease and Systemic Inflammation: Current Evidence

Heart failure is recognized as a condition characterized by chronic systemic inflammation, which contributes to cardiac remodeling, endothelial dysfunction, and clinical deterioration. In patients with HF with reduced ejection fraction (HFrEF) as well as those with preserved ejection fraction (HFpEF), persistently elevated levels of inflammatory markers are commonly observed, particularly CRP and pro-inflammatory interleukins. Although recent studies have explored the role of the gut microbiota in this process, the oral bacterial environment has also emerged as a relevant source of systemic inflammatory stimuli [8].

The presence of periodontitis has been consistently associated with elevated inflammatory biomarkers in patients with HF. In a population-based study, Chang et al. demonstrated that the absence of periodontal disease and the adoption of regular oral care practices, such as frequent toothbrushing and dental visits, were associated with a lower risk of atrial fibrillation and HF, whereas a greater number of missing teeth was linked to increased risk [32]. More directly, Molinsky et al. found that patients with periodontitis exhibited persistently higher CRP levels throughout follow-up [5], a finding further supported by Schmalz et al., who associated gingival bleeding and an increased periodontal inflamed surface area (PISA) with systemic infections and sepsis in individuals with chronic heart failure [24].

Findings from Walther et al. further showed that patients with severe periodontitis exhibited significantly higher concentrations of high-sensitivity C-reactive protein (hs-CRP) and interleukin-6, indicating that the severity of periodontal inflammation translates into greater systemic inflammatory activation [27]. These results are consistent with previous reviews identifying periodontitis as an important determinant of vascular and endothelial inflammation [28, 30]. Moreover, broader reviews of the literature emphasize that both genetic and inflammatory factors play a central role in linking periodontitis to systemic diseases, including cardiovascular conditions, suggesting a shared underlying susceptibility [33].

However, not all studies have confirmed this association. Aoyama et al. found no significant differences in serum inflammatory markers (CRP, HbA1c, lipids) between patients with high or low antibody levels against *P. gingivalis* [23]. Similarly, Dekker et al. identified only a moderate correlation between serum and salivary CRP, but no robust association with IL-6 or IL-10 [31]. These discrepancies may be attributed to methodological differences, variation in sample size, and the diagnostic criteria applied for both periodontal and cardiac conditions.

Evidence from interventional studies, however, suggests a stronger causal connection. Czerniuk et al. documented a significant reduction in plasma TNF-α and NT-proBNP levels following periodontal treatment in patients with CHF [26]. In another pilot study, the same authors observed a decrease in mean platelet volume (MPV), a marker of platelet activation, after periodontal intervention, suggesting that improvements in oral health may attenuate systemic inflammation and positively impact both hematological and cardiac parameters [34].

**Fig. 1:**
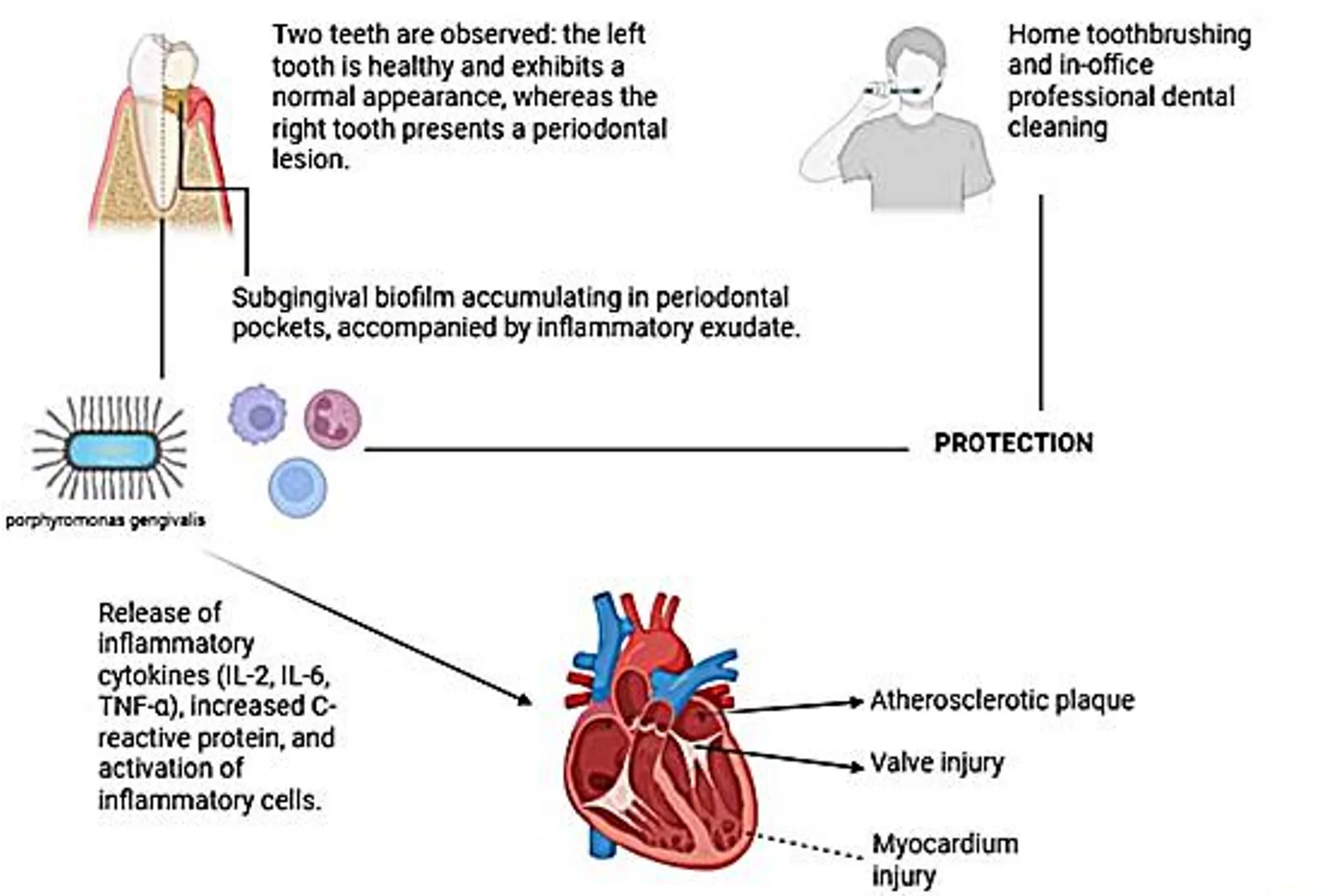
Association between periodontitis and cardiovascular outcomes. Subgingival biofilm accumulation in periodontal pockets, with microorganisms such as *Porphyromonas gingivalis*, triggers a local inflammatory response accompanied by exudate. The subsequent systemic inflammation promotes the release of pro-inflammatory cytokines (IL-2, IL-6, TNF-α), increased C-reactive protein, and activation of inflammatory cells, contributing to the development of atherosclerotic plaque, valvular lesions, and myocardial damage. Home oral hygiene combined with professional dental cleaning acts as a protective factor against these complications. Image created with BioRender

These lines of evidence indicate that periodontal disease has a relevant impact on systemic inflammation in patients with HF, contributing both to the elevation of inflammatory biomarkers and to the worsening of immune responses. Despite existing controversies, data from population-based cohorts, clinical studies, and recent narrative reviews converge on the notion that maintaining periodontal health may represent an adjunctive strategy to reduce systemic inflammation and potentially modify the prognosis of heart failure. As shown in Fig. 1.

### From Evidence to Perspectives: What Lies Ahead

Despite advances in understanding the relationship between periodontitis and HF, important methodological gaps remain. Most of the current evidence comes from observational and cohort studies, which indicate that periodontitis increases the risk of adverse cardiovascular events, including all-cause mortality [35, 36]. However, to confirm the causal direction of this association, larger randomized clinical trials are needed to evaluate the impact of periodontal treatment on clinically relevant outcomes in patients with HF.

In addition, the systemic effects of periodontal therapy have attracted increasing interest. Clinical trials and meta-analyses have demonstrated that non-surgical therapy can significantly reduce inflammatory markers such as interleukin-6 and CRP, as well as lower systolic blood pressure [16, 37]. Additional evidence suggests that periodontal treatment may also improve lipid profile and endothelial function, indicating a potential pathway for reducing cardiovascular risk [38, 39].

Another key point concerns the need for greater integration between cardiology and dentistry in clinical practice. Dental screening protocols for cardiac patients are still not part of routine hospital care, yet studies have shown that oral health education programs improve both periodontal and cardiovascular outcomes in high-risk individuals [40]. Narrative reviews have also emphasized that poor oral health is associated with atherosclerosis and cardiovascular events, but it remains insufficiently considered in daily cardiology practice [41].

Finally, from a public health perspective, there is growing recognition that preventive oral health strategies may positively influence cardiovascular risk. Recent data indicate that preventive dental care reduces infectious and cardiovascular complications in vulnerable populations, such as patients undergoing hemodialysis [39]. Therefore, public policies that promote access to dental care integrated into cardiovascular management may represent an effective strategy for large-scale health promotion.

## Conclusion and Future Directions

In conclusion, consistent evidence points to an association between periodontal disease and HF. Patients with periodontitis present with higher levels of inflammatory and cardiac biomarkers, such as CRP and NT-proBNP, suggesting that periodontal inflammation may contribute to the systemic inflammatory burden that characterizes HF. Interventional studies reinforce this link by demonstrating that periodontal treatment is associated with significant reductions in markers such as TNF-α and mean platelet volume, indicating a potential cardiovascular benefit from improvements in oral health.

In addition, cohort and population-based studies have shown a higher prevalence and incidence of heart failure among individuals with moderate to severe periodontitis, reinforcing the role of periodontal disease as a possible risk factor or modulator of cardiac dysfunction progression. However, it is important to acknowledge the limitations of the current literature. Many studies are limited by small sample sizes, lack of healthy control groups, or heterogeneous methodologies for diagnosing both periodontal disease and heart failure. The predominance of cross-sectional and observational studies makes it difficult to establish direct causality. Moreover, confounding factors such as metabolic comorbidities and lifestyle habits are not always uniformly controlled, which may influence the results.

Thus, although current findings support the hypothesis of a significant link between periodontitis and heart failure, large-scale longitudinal studies and randomized clinical trials are still needed. Such studies could not only confirm the causal direction of this association but also elucidate the underlying pathophysiological mechanisms. Consolidating this knowledge may pave the way for new preventive and integrated management strategies in which oral health is recognized as an essential component of cardiovascular care.

## Key References

- Schulze A, Kwast S, Pökel C, Busse M. Assessment of the relationship between periodontitis and cardiac parameters in patients with early chronic heart failure: a cross-sectional study. J Funct Morphol Kinesiol. 2024;9(1):52. https://doi.org/10.3390/jfmk9010052.
  - ○ This study directly evaluated the relationship between periodontitis and cardiac parameters in patients with chronic heart failure. Patients with periodontitis showed higher NT-proBNP and troponin T levels and lower left ventricular systolic and diastolic function parameters, providing clinically relevant evidence for the potential relationship between periodontal and cardiac conditions.
- Kolte AP, Kolte RA, Bawankar PV, Bajaj VA. Assessment and correlation of the influence of non-surgical periodontal therapy on serum lipid profile and cytokines in patients with stage III periodontitis. Int J Dent Hyg. 2023;21(2):298–304. https://doi.org/10.1111/idh.12639.
  - ○ This study demonstrated reductions in serum pro-inflammatory cytokines and improvements in lipid parameters following non-surgical periodontal therapy in patients with stage III periodontitis. These findings support the potential systemic effects of periodontal treatment and its relevance to cardiovascular risk-related pathways.
- Sharma S, Mathur D, Goswami S, et al. Prevalence of periodontitis in chronic heart failure patients and its relationship with NT-ProBNP: A cross-sectional study. Indian Heart J. 2025;77(2):110–3. https://doi.org/10.1016/j.ihj.2025.03.004.
  - ○ This study demonstrated a high prevalence of moderate and severe periodontitis among patients with chronic heart failure and identified an association between periodontal severity and elevated NT-proBNP levels in patients younger than 75 years, providing recent clinical evidence supporting the relationship between periodontal and cardiac status.

## Data Availability

No datasets were generated or analysed during the current study.

## Declarations

### Use of Artificial Intelligence

Artificial intelligence (AI) tools were used exclusively for language and grammar review. AI was not used to generate scientific content, analyze or interpret data, create figures or tables, or draw conclusions. All content was critically reviewed and approved by the authors, who take full responsibility for the final manuscript.
